# Author Correction: A Tunguska sized airburst destroyed Tall el-Hammam a Middle Bronze Age city in the Jordan Valley near the Dead Sea

**DOI:** 10.1038/s41598-023-35266-6

**Published:** 2023-05-22

**Authors:** Ted E. Bunch, Malcolm A. LeCompte, A. Victor Adedeji, James H. Wittke, T. David Burleigh, Robert E. Hermes, Charles Mooney, Dale Batchelor, Wendy S. Wolbach, Joel Kathan, Gunther Kletetschka, Mark C. L. Patterson, Edward C. Swindel, Timothy Witwer, George A. Howard, Siddhartha Mitra, Christopher R. Moore, Kurt Langworthy, James P. Kennett, Allen West, Phillip J. Silvia

**Affiliations:** 1 Geology Program, School of Earth and Sustainability, Northern Arizona University, Flagstaff, AZ 86011 USA; 2 Center of Excellence in Remote Sensing Education and Research, Elizabeth City State University, Elizabeth City, NC 27909 USA; 3 Department of Natural Sciences, Elizabeth City State University, Elizabeth City, NC 27909 USA; 4 Materials and Metallurgical Engineering, New Mexico Institute On Mining & Technology, Socorro, NM 87801 USA; 5 Los Alamos National Laboratory (Retired), Los Alamos, NM 87545 USA; 6 Analytical Instrumentation Facility, North Carolina State University, Raleigh, NC 27695 USA; 7 EAG Laboratories, Eurofins Materials Science, Raleigh, NC 27606 USA; 8 Department of Chemistry and Biochemistry, DePaul University, Chicago, IL 60614 USA; 9 Geophysical Institute, University of Alaska Fairbanks, 903 Koyukuk Drive, College, AK 99775 USA; 10 Faculty of Science, Charles University, Albertov 6, Prague, 12843 Czech Republic; 11 Southern Research Institute, 757 Tom Martin Drive, Birmingham, AL 35211 USA; 12 US Navy, NAVFAC Mid-Atlantic Region, NS Norfolk, VA 23511 USA; 13 Comet Research Group, Prescott, AZ 86301 USA; 14 Restoration Systems, L.L.C., Raleigh, NC 27604 USA; 15 Department of Geological Sciences, East Carolina University, Greenville, NC 27858 USA; 16 Savannah River Archaeological Research Program, South Carolina Institute of Archaeology and Anthropology, University of South Carolina, New Ellenton, SC 29809 USA; 17 CAMCOR, University of Oregon, 1443 E 13th Ave, Eugene, OR 97403 USA; 18 Department of Earth Science and Marine Science Institute, University of California, Santa Barbara, CA 93106 USA; 19 College of Archaeology, Trinity Southwest University, Albuquerque, NM 87109 USA

Correction to: *Scientific Reports* 10.1038/s41598-021-97778-3, published online 20 September 2021

The original version of this Article inaccurately described the work of Dr Mark Boslough presented in Figure 53, by suggesting that the simulation presented in this figure is of the Tunguska event. However, the simulated event was much larger in force than the Tunguska event. The discussion and legend of Figure 53 were revised as follows to reflect this.

As a result, in section ‘Analogous destruction events’, the title of sub-subsection ‘Comparison of TeH to the Tunguska airburst’ within ‘Hypothetical Tunguska‑class airburst near TeH ’ was removed. This paragraph was revised, where

“The damage at TeH appears similar to but higher than that of the well-documented airburst at Tunguska, Siberia in 1908. A supercomputer-generated model of a hypothetical 15-megaton airburst at Tunguska was developed at Sandia National Laboratories by Boslough^197^ (Fig. 53). He wrote that when a bolide explodes in the atmosphere, a high-temperature jet of ionized gases and impactor fragments reaches Earth’s surface at high velocity, excavates unconsolidated sediment, and expands radially outward in what is sometimes called a ‘base surge’. Surface temperatures rise higher than the melting points of silica-rich materials, and the surge’s radial velocity can exceed the speed of sound (1225 km/h or 761 mph). Radiative and convective heating can transform surface and excavated materials into meltglass^101^. Svetsov^162^ computer-modeled the airburst of an 80-m-wide impactor and found that radiative fluxes from the blast were sufficiently high to melt ~ 0.5 cm of surface sediment at > 1700 °C for a duration of ~ 20 s. This closely matches the half-centimeter-thick melting of mudbricks, pottery, and roofing clay observed TeH, making a hypothetical Tunguska-class airburst a plausible scenario. Even though the Sandia computer model has large uncertainties, the modeled scenario accounts for all the evidence, including the destruction of thick mudbrick walls at TeH and Jericho (Table 2).”

now reads:

“The damage at TeH appears similar to but higher than that of the ~ 5-megaton airburst at Tunguska, Siberia in 1908. A supercomputer-generated model of a hypothetical 15-megaton airburst was developed at Sandia National Laboratories by Boslough^197^ (Fig. 53) and shows conditions that are more energetic than for the 5-megaton Tunguska model also discussed by Boslough ^197.^ In our study, we propose two models for the TeH event that are also larger than estimated for Tunguska, a 12- and a 23-megaton model. For details, see Supporting Information, Tables S10–S11.

In summary, when a bolide as large as that proposed for TeH explodes in the atmosphere, we posit that a high-temperature jet reaches Earth’s surface at high velocity, excavates unconsolidated sediment, and expands radially outward in what is sometimes called a ‘base surge’. Surface temperatures rise higher than the melting points of silica-rich materials, and the surge’s radial velocity can exceed the speed of sound (1225 km/h or 761 mph). Radiative and convective heating can transform surface and excavated materials into meltglass^101^. Svetsov^162^ computer-modeled the airburst of an 80-m-wide impactor and found that radiative fluxes from the blast were sufficiently high to melt ~ 0.5 cm of surface sediment at > 1700 °C for a duration of ~ 20 s. This closely matches the half-centimeter-thick melting of mudbricks, pottery, and roofing clay observed TeH, making it possible that an airburst occurred that was larger than at Tunguska. The modeled scenarios account for all the evidence, including the destruction of thick mudbrick walls at TeH and Jericho (Table 2).”

Additionally, Figure 53 was updated with a version that does not contain any labels. The original version is included below for the record.

**Figure 53:**
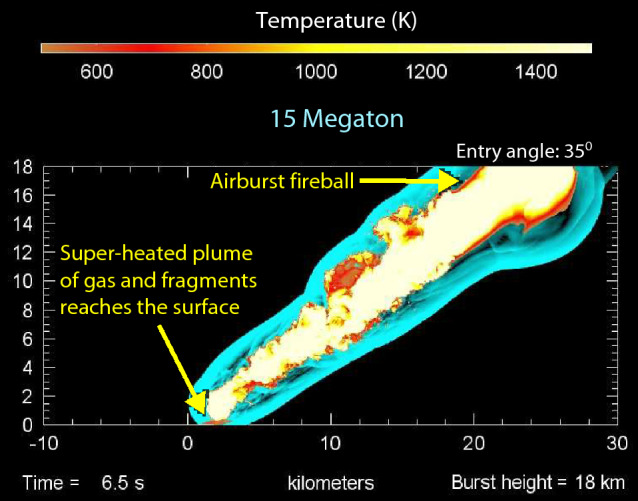
Supercomputer 15-megaton model of the Tunguska airburst. For the computer calculations of the airburst model, the entry angle is 35° and the detonation height is 18 km. After 6.5 s, the airburst width at the top is ~ 6 km. The hypervelocity jet of ionized gases and impactor fragments is ~ 2 km wide at the surface of the ground and expands at 1225 km/h (761 mph). The temperature scale at the top ranges from ~ 500° to 1500° K. Conference presentation slide 26 from Boslough^197^, Sandia National Laboratories (US Department of Energy) is in the public domain.

Finally, the legend of Figure 53 was revised.

“Supercomputer 15-megaton model of the Tunguska airburst. For the computer calculations of the airburst model, the entry angle is 35° and the detonation height is 18 km. After 6.5 s, the airburst width at the top is ~ 6 km. The hypervelocity jet of ionized gases and impactor fragments is ~ 2 km wide at the surface of the ground and expands at 1225 km/h (761 mph). The temperature scale at the top ranges from ~ 500° to 1500° K. Conference presentation slide 26 from Boslough^197^, Sandia National Laboratories (US Department of Energy) is in the public domain.”

now reads:

“Supercomputer 15-megaton model of an airburst larger than the one at Tunguska. For the computer calculations of the airburst model, the entry angle is 35° and the detonation height is 18 km. At 6.5 s, the near-surface temperatures are at the high end of the temperature scale that ranges up to >1400° K. Conference presentation slide 26 from Boslough^197^, Sandia National Laboratories (US Department of Energy) is in the public domain.”

The Article has been corrected.

